# Supplementary material for: WordCommentsAnalyzer: A windows software tool for qualitative research
Source: F1000Res. 2018 Sep 4;7:536. Originally published 2018 May 3. [Version 2] doi: 10.12688/f1000research.14819.2 (PMC6137411; doi:10.12688/f1000research.14819.2)
Supplement: Supplementary file 3 [file f1000research-7-17614-s0002.tgz › b2a79496-4ada-47c4-9682-a99ce867836d.zip!/Eight Iranian Health Journals/Journal of Education and Community Health_12.docx]

© Journal of Education and Community Health. This work is licensed under CC BY-NC 4.0 (https://creativecommons.org/licenses/by-nc/4.0/).

Volume & Issue: دوره 2 - شماره 1

Title: تاثیر مشاوره ورزشی بر مقیاس های عملکردی کیفیت زندگی زنان مبتلا به سرطان پستان

http://jech.umsha.ac.ir/article-1-113-fa.html

سابقه و هدف: سرطان پستان یکی از شایع ترین سرطان ها و کشنده ترین بدخیمی در میان زنان در دنیاست. تشخیص، درمان و عوارض ناشی از آن سبب بروز آثار و عوارض متعدد جسمی، روانی، اجتماعی و کاهش کیفیت زندگی این بیماران می گردد. هدف از این پژوهش تعیین تاثیر مشاوره ورزشی بر مقیاس های عملکردی زنان مبتلا به سرطان پستان می باشد.  مواد و روش ها: پژوهش حاضر یک مطالعه نیمه تجربی دو مرحله ای قبل و بعد از مداخله با دو گروه مداخله (مشاوره ورزشی) و کنترل است که در آن 60 زن مبتلا به سرطان پستان مراجعه کننده به مراکز درمانی بیماران سرطانی شهر همدان با روش نمونه گیری تصادفی در دو گروه مداخله (30 نفر) و کنترل (30نفر) قرار گرفتند. مداخله شامل 2 هفته مشاوره ورزشی در سالن ورزشی(هرهفته 2 جلسه و هر جلسه 60-45 دقیقه) و 8 هفته اجرای تمرینات ورزشی در منزل که از آبان 1393 تا اسفند 1393 انجام شد. در طی این مدت گروه کنترل فقط اقدامات درمانی روتین دریافت نمودند. داده ها با استفاده از پرسشنامه اطلاعات دموگرافیک؛ پرسشنامه های QLQ-C30 و QLQ BR-23 جمع آوری و تکمیل گردید.داده ها با نرم افزار spss.20 و آزمون های کای دو،فیشر، تی تست و ANCOVA تجزیه و تحلیل شد.  یافته ها: نتایج نشان داد که نمره کلی حیطه عملکردی در گروه مداخله قبل از مداخله 19.27±61.81 و بعد از مداخله 9.25±86.91 بود که تفاوت آماری معناداری وجود داشت (P<0.05). بیشترین تغییرات در حیطه عملکرد جسمانی، عملکرد احساسی، و تصویر بدنی بود. در حیطه های عملکرد شناختی، اجتماعی، جنسی، لذت جنسی و دورنمای آینده پس از مداخله در دو گروه تفاوت معناداری وجود نداشت (P>0.05).  نتیجه گیری: مشاوره ورزشی بر حیطه های عملکردی کیفیت زندگی زنان مبتلا به سرطان پستان اثر مثبت داشته و باعث بهبودی قابل توجهی در این بیماران می گردد. این روش می تواند به عنوان بخشی از اقدامات روتین این بیماران در کنار سایر اقدامات درمانی قرار گیرد.

Title: پیشگویی کننده های مصرف دخانیات در دانشجویان علوم پزشکی اهواز: مطالعه ای بر اساس نظریه رفتار برنامه ریزی شده

http://jech.umsha.ac.ir/article-1-81-fa.html

سابقه و هدف: مصرف دخانیات توسط دانشجویان علوم پزشکی نقش مهمی در سلامت کل جامعه ایفا می نماید، چرا که این گروه در رفتارهای مرتبط با سلامت نقش الگو را دارند. مطالعه حاضر با هدف تعیین پیشگویی‌کننده‌های مصرف دخانیات در دانشجویان علوم پزشکی اهواز با استفاده از نظریه رفتار برنامه‌ریزی‌شده انجام شده است. مواد و روش‌ها: در این مطالعه مقطعی170 دانشجوی علوم پزشکی اهواز (56 پسر و 114 دختر) به روش در دسترس انتخاب شدند. نمونه‌ها پرسشنامه‌ای شامل مشخصات جمعیت‌شناختی و سازه‌های نظریه رفتار برنامه‌ریزی‌شده را تکمیل نمودند. آزمون رگرسیون لجستیک برای محاسبه نسبت شانس (در فاصله اطمینان 95%) عوامل جمعیت‌شناختی و سازه‌های نظریه رفتار برنامه‌ریزی‌شده در پیشگویی رفتار مصرف دخانیات استفاده شد. اطلاعات جمع‌آوری ‌شده با استفاده از ویرایش 18 نرم‌افزار SPSS تجزیه و تحلیل شد. یافته‌ها: در مجموع 36 درصد از پسران و 3.5 درصد از دختران مصرف کننده فعلی دخانیات بودند. مدل رگرسیون لجستیک نشان داد که مرد بودن، داشتن دوستان سیگاری، والدین سیگاری و داشتن برادر سیگاری با مصرف دخانیات مرتبط بود. همچنین همه سازه‌های نظریه رفتار برنامه‌ریزی‌شده شامل نگرش، نرم‌های انتزاعی، کنترل رفتاری و قصد پیشگویی‌کننده معنی‌دار مصرف دخانیات بودند. نتیجه‌گیری: بر اساس مطالعه حاضر نرم‌های انتزاعی و دوستان سیگاری مهم‌ترین پیشگویی‌کننده‌های مصرف دخانیات در دانشجویان علوم پزشکی بودند.

Title: نقش سلامت معنوی و حمایت اجتماعی درپیش بینی کیفیت زندگی پرستاران

http://jech.umsha.ac.ir/article-1-105-fa.html

زمینه و هدف: سلامت معنوی ابعاد مختلف زندگی را با همدیگر هماهنگ کرده و کارکرد روانی و سازگاری را تقویت می کند. کیفیت زندگی می تواند تحت تاثیر عوامل روحی-روانی و محیط های شغلی قرار گیرد. این پژوهش با هدف ارزیابی نقش حمایت اجتماعی و سلامت معنوی در کیفیت زندگی پرستاران انجام شد.  مواد و روش ها: این مطالعه توصیفی-تحلیلی در سال۱۳۹۴ بر روی پرستاران زن شاغل (۲۰۰ نفر) در شهر همدان انجام شد. از بین بیمارستانهای آموزشی، دو بیمارستان به صورت تصادفی انتخاب و به روش سرشماری کلیه پرستاران مورد بررسی قرار گرفتند. داده ها با استفاده از پرسشنامه کیفیت زندگی سازمان بهداشت جهانی، پرسشنامه های حمایت اجتماعی و بهزیستی معنوی پالوتزین و الیسون جمع آوری گردید. داده ها با استفاده از آزمون رگرسیون چندگانه و به وسیله نرم افزار SPSS-18 مورد تجزیه و تحلیل قرار گرفت .  یافته ها: میانگین سنی افراد تحت پژوهش 6.63±33.86 سال بود. نتایج تحلیل رگرسیون نشان داد که از بین مولفه های حمایت اجتماعی سازه های حمایت عاطفی ، ابزاری و اطلاعاتی سهم معنی داری در پیش بینی کیفیت زندگی داشتند و همچنین از بین سازه های سلامت معنوی تنها سازه سلامت مذهبی بر کیفیت زندگی پرستاران اثر معنی داری داشت (P<0.05).  نتیجه گیری: طبق یافته ها به نظر می رسد انجام مداخلات آموزشی و ارائه بسته های آموزشی جهت افزایش سلامت معنوی و ارتقاء کیقیت زندگی پرستاران ضروری باشد.

Title: تعیین ارتباط ابعاد مختلف حمایت اجتماعی با کیفیت زندگی سالمندان منطقه 8 شهر تهران در سال 1392

http://jech.umsha.ac.ir/article-1-102-fa.html

سابقه و هدف: از جمله مسائل مهم زندگی سالمندان کیفیت زندگی و عوامل مهم موثر بر آن همچون حمایت اجتماعی می باشد چرا که سالمندان از گروه های آسیب پذیر جامعه بوده و برای تأمین کیفیت زندگی باید زیر پوشش حمایت های گوناگون قرار بگیرند. هدف این مطالعه تعیین ارتباط ابعاد مختلف حمایت اجتماعی بر کیفیت زندگی سالمندان منطقه 8 شهر تهران می باشد. مواد و روش ها: این پژوهش یک مطالعه مقطعی می باشد که بر روی 240 سالمند بالای 60 سال ساکن در منطقه 8 شهر تهران که به روش نمونه گیری خوشه ای انتخاب شدند، انجام گردید. برای سنجش کیفیت زندگی از پرسشنامه کیفیت زندگی لیپاد و برای اندازه گیری حمایت اجتماعی از پرسشنامه حمایت اجتماعی نوربک استفاده شد. داده ها با استفاده از نرم افزار SPSS-20 و آزمون های آماری من ویتنی و کروسکال والیس و آزمون همبستگی اسپیرمن تجزیه و تحلیل گردید. یافته ها: میانگین نمره کیفیت زندگی (از 93 نمره) در سالمندان مرد 68.9 و در زنان 67.6 بود که این تفاوت از نظر آماری معنی دار نبود (P=0.404). متغیر تحصیلات بر کیفیت زندگی و نیز حمایت اجتماعی تأثیرگذار بوده که از نظر آماری معنی-دار می باشد (P<0.05). بین انواع حمایت اجتماعی با عملکرد فیزیکی، ذهنی، اجتماعی و رضایت از زندگی ارتباط آماری معنی دار وجود دارد (P<0.05). نتیجه گیری: انواع حمایت اجتماعی در مردان بیشتر از زنان است. اگرچه ارتباط بین ابعاد حمایت اجتماعی با نمره کلی کیفیت زندگی از نظرآماری معنی دار نبوده اما ارتباط آماری معنی‌دار ابعاد حمایت اجتماعی با ابعاد کیفیت زندگی حاکی از تأثیر حمایت اجتماعی بر کیفیت زندگی می‌باشد.

Title: کاربرد مدل بزنف در تحلیل رفتار خوددرمانی در بین بیماران دیابتی نوع دو شهر کرمانشاه در سال 1393

http://jech.umsha.ac.ir/article-1-67-fa.html

سابقه و هدف: خوددرمانی در بیماران دیابتی به سبب به هم زدن تعادل قند خون باعث ایجاد عوارض خطرناک و کشنده‌ای در آن‌ها می‌گردد. لذا مطالعه حاضر با هدف بررسی رفتار خوددرمانی در بیماران دیابتی نوع دوم با بهره‌گیری از مدل بزنف انجام گردید. مواد و روش‌ها: این مطالعه توصیفی– تحلیلی در سال 1393 بر روی 380 نفر از بیماران دیابتی نوع دو مراجعه‌کننده به کلینیک مرکزی دیابت شهر کرمانشاه انجام گردید. آزمودنی‌ها به روش نمونه‌گیری تصادفی ساده انتخاب و وارد مطالعه شدند. پرسشنامه مشتمل بر اطلاعات جمعیت شناختی و سازه‌های الگوی بزنف بود. داده‌ها پس از جمع‌آوری از طریق مصاحبه با استفاده از نرم‌افزار SPSS-16 و بهره‌مندی از آزمون‌های آماری من ویتنی، کورسکال والیس و رگرسیون لجستیک و کای دو تجزیه و تحلیل گردید. یافته‌ها: در این مطالعه 50.5 درصد بیماران دیابتی دارای رفتار خوددرمانی بودند. طبق یافته‌ها سازه‌های الگوی بزنف توانست 24 درصد از بسامد رفتارهای خوددرمانی را تبیین نماید. همچنین در مدل رگرسیون لجستیک سازه‌های نگرش، هنجارهای انتزاعی و عوامل قادر کننده سهم معنی‌داری در پیش‌بینی رفتارهای خوددرمانی داشتند (P<0.05). نتیجه‌گیری: یافته‌های مطالعه حاضر حاکی از شیوع بالای خوددرمانی در بین بیماران دیابتی نوع 2 بود. به نظر می‌رسد کار گیری الگوی بزنف می‌تواند ابزاری اثربخش برای پیش‌بینی رفتارهای خوددرمانی در بیماران دیابتی باشد.

Title: بررسی مقایسه ای تأثیر آموزش از طریق نرم افزار چند رسانه ای و آموزش چهره به چهره برمیزان آگاهی مادران باردار از علائم خطردوران بارداری و پس از زایمان

http://jech.umsha.ac.ir/article-1-103-fa.html

سابقه و هدف: افزایش آگاهی مادران باردار در مورد علائم خطر دوران بارداری و پس از زایمان یکی از عوامل مهم در کاهش مرگ ومیر مادران می باشد. همچنین بکارگیری روش آموزش مناسب می تواند روندآموزش را تحت تأثیر قرار دهد. هدف از این مطالعه تعیین تأثیر آموزش از طریق نرم افزار چند رسانه ای و آموزش چهره به چهره بر میزان آگاهی مادران باردار از علائم خطر دوران بارداری و پس از زایمان بوده است. مواد و روش ها: این مطالعه مداخله ای و از نوع تجربی می باشد. در این پژوهش 120 زن نخست زای مراجعه کننده به مراکز بهداشتی درمانی شهرستان کرج به روش نمونه گیری آسان انتخاب و با تخصیص تصادفی در دو گروه قرار گرفتند. گروه اول به صورت چهره به چهره و گروه دوم از طریق دیسک فشرده- CD -آموزش علائم خطر را دریافت نمودند. ده هفته پس از مداخله پس آزمون انجام گرفت و در نهایت اطلاعات به دست آمده با استفاده از آزمون های آماری مورد تجزیه و تحلیل قرار گرفت. یافته ها: 10 هفته پس از مداخله میانگین امتیاز آگاهی بین دو گروه آموزشی اختلاف آماری معنا داری نداشت ، گرچه مقایسه امتیاز آگاهی در هر یک از گروه ها قبل و پس از مداخله تفاوت آماری معنا داری را نشان می دهد (P<0.001). نتیجه گیری: آموزش از طریق نرم افزار چند رسانه ای و آموزش چهره به چهره هر دو به یک میزان درارتقای آگاهی مادران بارداردر مورد علائم خطر موثر بوده است. با در نظر گرفتن این موضوع و نیز با توجه به محدودیت زمانی پرسنل مراکز بهداشتی درمانی برای آموزش چهره به چهره، به نظر می رسد استفاده از نرم افزار جایگزین خوبی برای آموزش چهره به چهره خواهد بود.

Title: تاثیر آموزش مهارت پخت غذای سالم در والدین بر وضعیت سلامت کودکان و نوجوانان: یک مرور ساختار مند

http://jech.umsha.ac.ir/article-1-72-fa.html

سابقه و هدف: پژوهش حاضر، مداخلات انجام شده در زمینه آموزش مهارت پخت غذا به والدین را به منظور شناسایی موثرترین روش آموزشی جهت ایجاد پیامدهای مثبت و طولانی اثر در ارتقاء سلامت کودکان و نوجوانان، به شیوه ساختارمند مورد بررسی و تحلیل قرار داده است.  مواد و روش ها: جستجوی الکترونیکی پایگاههای اطلاعاتی با استفاده از کلمات کلیدی فارسی و انگلیسی انجام شد. پایگاههـای مـورد بررسـی عبـارت بودند ازSCOPOUS, PROQUEST SCIENCE DIRECT, PUBMED, SPRINGER ,SID , Iran Medex, Google Scholar. مطالعات مداخله آموزشی که شامل افزایش دانش و مهارت پخت غذای سالم در والدین، همراه با سنجش تاثیر مداخله بر کودکان و نوجوانان بوده و بین ژانویه 2005 تا سپتامبر 2014 به چاپ رسیده بودند، مورد بررسی قرار گرفتند.  یافته ها: مداخلات در زمینه آموزش مهارت پخت غذای سالم به والدین به دو دسته کلی (الف) مبتنی بر تئـوری هـا و مـدل هـای آمـوزش بهداشـت و (ب) مطالعات بـدون استفاده از مدلها و تئوریهای آموزش بهداشت تقسیم شدند. از 7 مقاله بررسی شده 4 مطالعه در دسته اول و 3 مطالعه در دسته دوم طبقه بندی گردیدند. مطالعات بر اساس ایجاد تغییر مطلوب در نمایه توده بدنی و همچنین ماندگاری تغییر رفتار انجام شده در پیگیری های بعدی مقایسه گردیدند.   نتیجه گیری: برنامه های آموزشی چند ترکیبی بهمراه استفاده از نظریه ها و مدل های آموزش بهداشت مؤثر، بهترین شیوه برای دستیابی به نتایج مورد انتظار می باشد. جهت ایجاد تغییرات مطلوب در نمایه توده بدنی کودکان و نوجوانان نیاز به انجام کارآزمایی های بالینی طولانی مدت با چندین استراتژی مختلف وجود دارد.
